# Concordance of Preoperative Transvaginal Ultrasound and Intraoperative Findings in Fibroid Ablation Using Transcervical Ultrasound: A Two‐Center Cohort Study

**DOI:** 10.1002/jum.70288

**Published:** 2026-05-20

**Authors:** Philip G. Hepp, Markus Fleisch, Ines Beyer

**Affiliations:** ^1^ Department of Obstetrics and Gynecology Universität Witten/Herdecke, KJF Klinik Josefinum gGmbH Augsburg Germany; ^2^ Department of Obstetrics and Gynecology Universität Witten/Herdecke, Universitätsklinikum Wuppertal Wuppertal Germany; ^3^ Department of Obstetrics and Gynecology Heinrich Heine Universität Düsseldorf, Klinikum Leverkusen Leverkusen Germany

**Keywords:** diagnostic concordance, minimally invasive surgery, transcervical radiofrequency ablation, transvaginal ultrasound, uterine fibroids

## Abstract

**Objective:**

To evaluate concordance between the number of uterine fibroids deemed clinically significant on preoperative transvaginal sonography (TVS) and the number of fibroids treated intraoperatively during transcervical radiofrequency ablation (RFA) under intrauterine ultrasound guidance.

**Methods:**

This is a retrospective, paired‐sample cohort study that is conducted in two tertiary care centers. Sixty‐seven women underwent transcervical RFA for symptomatic uterine fibroids. This study compares TVS‐significant with intraoperatively treated fibroid counts.

**Results:**

TVS identified a mean of 1.60 ± 0.94 clinically significant fibroids, whereas 1.90 ± 1.23 were treated intraoperatively (mean paired difference + 0.30; 95% CI 0.11–0.49, *P* = .0036). Intraoperative counts exceeded TVS in 28%, were identical in 66%, and were lower in 6% of patients. Categorized exact agreement was 73.1% with substantial concordance (weighted *κ* = 0.61; 95% CI 0.42–0.76). In the a priori multifibroid subgroup (≥2 clinically significant fibroids on TVS; n = 23), the mean paired difference (+0.26) was not statistically significant (*P* = .266). Treated fibroid count correlated strongly with TVS count (*ρ* = 0.72, *P* < .001) and modestly with ablation time (*ρ* = 0.26, *P* = .033), but not with postoperative changes in hemoglobin or inflammatory markers (all *P* > .10).

**Conclusion:**

Preoperative TVS demonstrates substantial diagnostic agreement with the fibroid burden treated during transcervical RFA. While TVS may numerically underrepresent the actionable fibroid count in a subset, systematic underestimation is not confirmed in multifibroid disease. The potential for intraoperative expansion should be considered during procedural planning.

**Abbreviations**
FIGO: International Federation of Gynecology and Obstetrics
RFA: radiofrequency ablation
TVS: transvaginal sonography

Uterine fibroids (leiomyomas) are the most common benign tumors of the female reproductive tract, with population‐based reviews estimating a lifetime prevalence of 70% or more.^1^, ^2^ Although many lesions remain clinically silent, symptomatic fibroids impose a substantial burden through heavy menstrual bleeding, pelvic pressure or pain, anemia, and fertility impairment. These symptoms translate into reduced quality of life and considerable healthcare costs, often driving patients toward invasive therapy and sometimes hysterectomy.^3^

Transvaginal ultrasound (TVS) is the first‐line imaging modality for diagnosing and mapping fibroids because it is widely available, inexpensive, and well tolerated. Its overall sensitivity for detecting fibroids approaches 90–99% when histology or MRI is used as the reference standard.^4^ However, TVS loses precision in uteri with large volume or multiple lesions and may under‐report clinically relevant submucosal components, thereby compromising surgical planning.^5^ A reliable estimate of “clinically significant” fibroid burden is essential for counseling, deciding on uterine‐sparing therapy, and setting realistic expectations for symptom control.

Minimally invasive, uterus‐preserving treatments have expanded rapidly in the last decade. Among these, transcervical radiofrequency ablation (RFA) under intrauterine ultrasound guidance has emerged as a promising alternative to hysterectomy. The Sonata® system (Hologic, Inc., Marlborough, MA, USA) integrates a steerable RF handpiece with a real‐time intrauterine ultrasound probe, enabling targeted ablation of individual fibroids under direct visualization.^6^ Prospective trials and registries have shown durable symptom relief, substantial fibroid‐volume reduction, and low reintervention rates up to 3 years post‐procedure.^7^, ^8^, ^9^ Unlike preoperative TVS, intraoperative mapping during transcervical RFA defines the therapeutically actionable fibroid burden that ultimately dictates the procedural strategy.

Despite these advances, the diagnostic concordance between TVS and intraoperative findings remains incompletely defined, particularly in patients selected for transcervical RFA. A discrepancy between preoperative imaging and intraoperative therapeutic decisions may influence procedural planning and expected procedural complexity.

Therefore, the present retrospective two‐center cohort study was designed to quantify the discrepancy between the number of fibroids classified as clinically significant on preoperative TVS and the number of fibroids actually treated during transcervical RFA. Secondary objectives were to evaluate whether discrepancies are systematic in patients with multiple lesions and to explore clinical correlates such as operative time and postoperative hemoglobin change. By clarifying the diagnostic reliability of TVS in this setting, our findings aim to inform patient counseling and set realistic procedural expectations.

## Methods

This retrospective study was conducted at two hospital centers for minimally invasive gynecology (KJF Klinik Josefinum, Augsburg, and Klinikum Leverkusen, Germany). The protocol was reviewed and approved by the institutional review board (IRB No. 001‐2025); the requirement for informed consent was waived because only de‐identified data were analyzed.

All consecutive patients undergoing transcervical RFA for symptomatic uterine fibroids at either center between December 1, 2024 and July 31, 2025 were included. Inclusion required documentation of the number of fibroids deemed clinically significant on preoperative TVS and the number of fibroids treated intraoperatively. Postoperative laboratory values (hemoglobin, leukocytes, and CRP) on postoperative days 1 and 2 were extracted when available. Sixty‐seven patients met these inclusion criteria.

Transvaginal ultrasound examinations were performed by fellowship‐trained gynecological sonographers using high‐resolution 2D transvaginal transducers (GE IC9‐RS vaginal probe, GE Health Care, Chicago, IL, USA). For each fibroid, size, location, and International Federation of Gynecology and Obstetrics (FIGO) type were recorded. All fibroids of FIGO type 0, 1, and 2 were labeled “TVS‐significant.” Additionally, fibroids judged by the attending gynecologist to be clinically relevant for symptoms or fertility were labeled “TVS‐significant.” Ultrasound reports followed a standardized template to enhance reproducibility.^5^

### Surgical Procedure

All procedures were carried out under general anesthesia in lithotomy position. After transcervical introduction of the Sonata handpiece, intrauterine ultrasound was used to create a real‐time fibroid map. Each fibroid was treated with one or more RF applications according to device algorithms. The number of fibroids deemed clinically significant intraoperatively (“intraoperative count”) and total RF energy time (seconds) were documented in the operative record.

For each patient, the following derived measures were computed: Hb_Diff = Hb(day +1) − Hb(day +2); Leuko_Diff = Leuko(day +2) − Leuko(day +1); CRP_Diff = CRP(day +2) − CRP(day +1).

Because fibroid counts are discrete and paired differences were non‐normally distributed, paired comparisons of TVS‐significant versus intraoperatively treated counts were performed using the Wilcoxon signed‐rank test (two‐sided). Associations between fibroid counts and procedural or laboratory variables were assessed using Spearman rank correlation with pairwise deletion for missing data. Analyses were conducted using IBM SPSS Statistics v23; *P* < .05 was considered statistically significant.

Concordance between TVS‐significant and intraoperatively treated fibroid counts was assessed using exact agreement percentages and Cohen's linearly weighted kappa for categorized counts (1, 2, and ≥3 fibroids). While Spearman rank correlation was calculated to assess the monotonic association between the counts, this does not measure absolute diagnostic agreement.

Furthermore, a subgroup analysis was performed in patients presenting with multifibroid disease, strictly defined a priori as ≥2 clinically significant fibroids on preoperative TVS. This approach was chosen to evaluate the discrepancy in more complex cases.

## Results

### Patient Characteristics

A total of 67 patients met all inclusion criteria and were included in the final analysis. The mean age was 44.4 ± 5.9 years, and the mean body mass index was 25.7 ± 4.5 kg/m^2^. Abnormal uterine bleeding was the primary indication for treatment in the vast majority of cases (86.6%), followed by bulk‐related symptoms. A potential desire for future fertility was reported by 25.4% of the patients. Regarding baseline fibroid burden, 23 patients (34.3%) were identified a priori with multifibroid disease (≥2 clinically significant fibroids on preoperative TVS), whereas 44 patients (65.7%) presented with a single clinically significant fibroid on initial imaging (Table 1).

**Table 1:** Baseline Characteristics and Perioperative Parameters of the Combined Cohort

Characteristic	Overall (N = 67)
Study center	
Josefinum, n (%)	32 (47.8%)
Leverkusen, n (%)	35 (52.2%)
Demographics	
Age at procedure, years	44.6 [41.2–48.8] 44.4 ± 5.9 Range 32.2–53.6 n = 32
Body mass index, kg/m^2^	24.2 [21.8–29.1] 25.7 ± 4.5 Range 18.7–35.6 n = 33
Height, cm	166.5 [162.0–170.0] 165.6 ± 5.9 Range 153.0–176.0 n = 34
Weight, kg	69.0 [61.0–77.0] 70.7 ± 13.7 Range 45.0–99.0 n = 33
Obstetric and gynecologic history	
Gravida (G)	2.0 [0.0–3.0] 1.6 ± 1.6 Range 0.0–6.0 n = 64
Para (P)	1.0 [0.0–2.0] 1.1 ± 1.2 Range 0.0–4.0 n = 64
Prior gynecologic surgery	Yes: 24 (35.8%) No: 19 (28.4%) Missing: 24
Potential desire for future fertility	Yes: 17 (25.4%) No: 50 (74.6%) Missing: 0
Symptoms	
Primary symptom (AUB versus other)	AUB: 58 (86.6%) Other: 5 (7.5%) Missing: 4
VAS bleeding (if available)	9.0 [8.0–10.0] 8.7 ± 1.7 Range 3.0–10.0 n = 20
VAS pain (if available)	4.0 [1.0–7.0] 4.3 ± 3.3 Range 0.0–9.0 n = 21
Fibroid burden and procedural parameters	
TVS fibroids (total), count	1.00 [1.00–2.00] 1.85 ± 1.16 Range 1.00–5.00 n = 67
TVS fibroids (clinically significant), count	1.00 [1.00–2.00] 1.60 ± 0.94 Range 1.00–4.00 n = 67
Intraoperatively treated fibroids, count	1.00 [1.00–2.00] 1.90 ± 1.23 Range 1.00–7.00 n = 67
Total RF ablation time, seconds	513 [261–714] 515 ± 315 Range 15–1314 n = 66
Postoperative laboratory values	
Hemoglobin day +1, g/dL	11.50 [10.35–12.25] 11.22 ± 1.57 Range 5.30–13.80 n = 66
Hemoglobin day +2, g/dL	11.30 [10.50–12.20] 11.23 ± 1.61 Range 5.60–14.30 n = 63
Hemoglobin difference (day +1 − day +2), g/dL	−0.10 [−0.40–0.35] −0.07 ± 0.57 Range − 1.40–1.30 n = 63
Leukocytes day +1, 10^3^/μL	9.00 [7.12–10.25] 9.23 ± 2.82 Range 4.10–16.80 n = 66
Leukocytes day +2, 10^3^/μL	6.70 [5.70–8.10] 6.96 ± 1.94 Range 3.50–11.80 n = 63
Leukocyte difference (day +2 − day +1), 10^3^/μL	−2.50 [−3.40–‐1.25] −2.31 ± 2.23 Range − 6.80–4.80 n = 63
CRP day +1, mg/L	0.59 [0.14–1.38] 1.44 ± 2.53 Range 0.00–14.20 n = 66
CRP day +2, mg/L	0.49 [0.10–1.10] 1.67 ± 3.87 Range 0.00–18.90 n = 63
CRP difference (day +2 − day +1), mg/L	−0.06 [−0.38–0.00] 0.21 ± 2.01 Range −1.77–10.20 n = 63

Data are shown as median [IQR], mean ± SD, range, and number of available observations (n) for continuous variables, and as n (%) for categorical variables. Postoperative laboratory values refer to day +1 and day +2 after the procedure.

### Primary Outcome: TVS Versus Intraoperative Fibroid Count

Across the entire cohort, the mean number of fibroids deemed clinically significant on TVS was 1.60 ± 0.94, whereas 1.90 ± 1.23 fibroids were treated intraoperatively using transcervical RFA.

**Figure 1:**
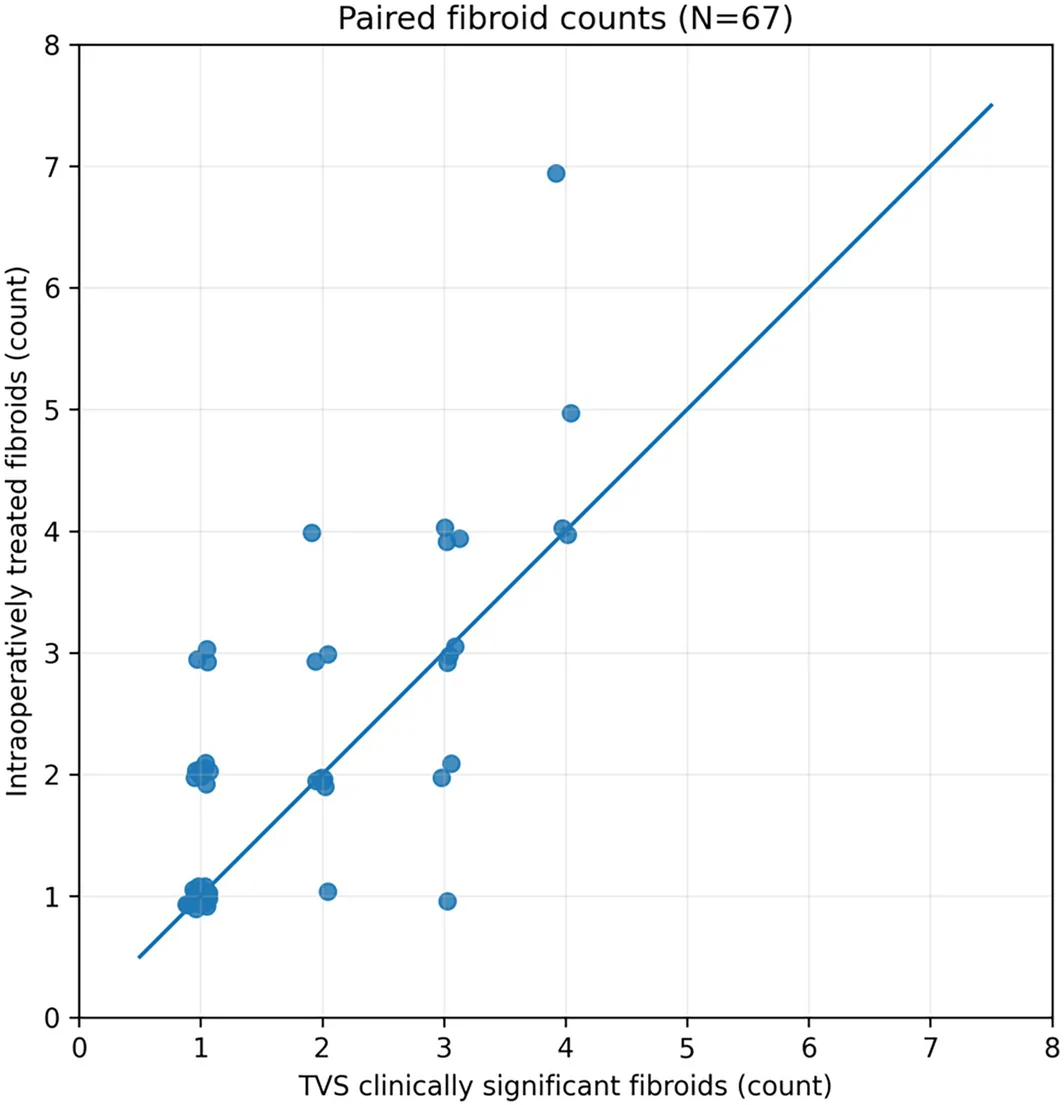
Paired fibroid counts on preoperative TVS versus intraoperatively treated fibroids. Scatterplot comparing the number of fibroids deemed clinically significant on preoperative transvaginal sonography (TVS) (*x*‐axis) with the number of fibroids treated intraoperatively by transcervical RFA (*y*‐axis) (N = 67). The solid line indicates identity (*y* = *x*). Points above the line represent patients in whom more fibroids were treated intraoperatively than classified as clinically significant on TVS. A small random jitter was applied to improve visualization of overlapping observations.

The paired difference (intraoperative minus TVS) had a median of 0 (IQR, 0 to 1) and a mean of +0.30 ± 0.78 fibroids (95% CI +0.11 to +0.49), indicating a statistically significant undercounting by preoperative TVS (Wilcoxon signed‐rank *P* = .0036) (Figure 1 and online supplemental Figure 1).

Directionally, intraoperative counts exceeded TVS counts in 19 patients (28%), were identical in 44 (66%), and were lower than TVS counts in 4 (6%).

**Figure 2:**
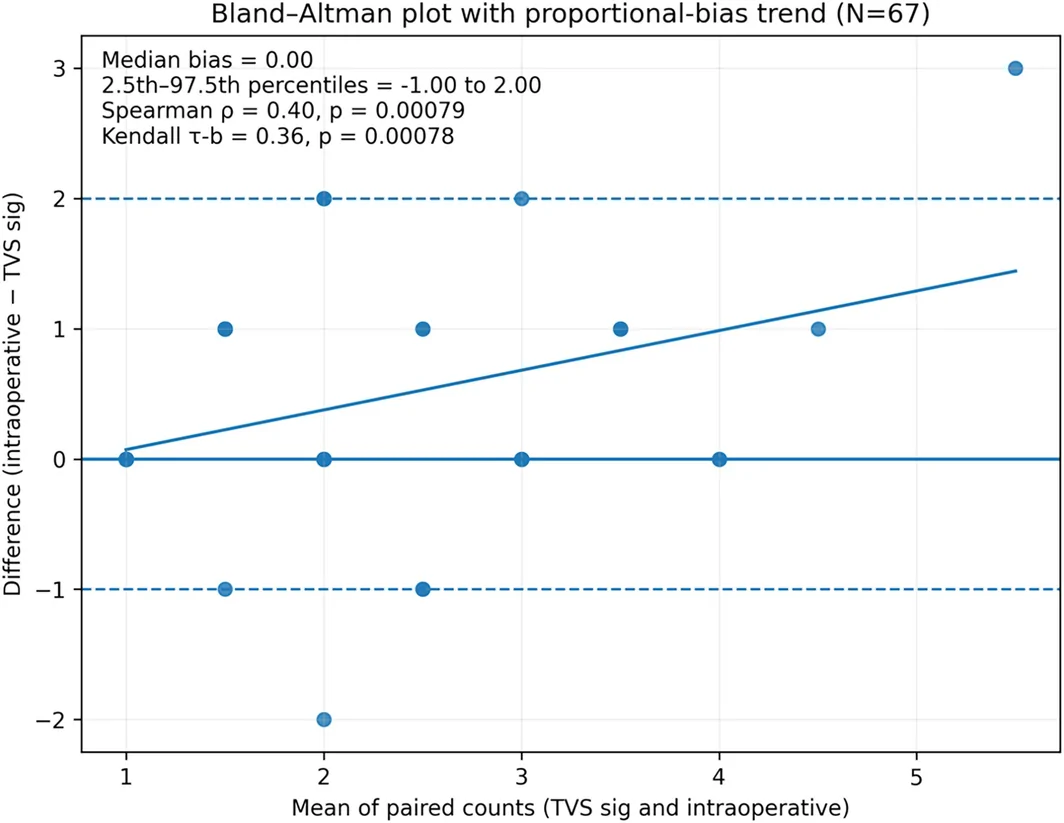
Bland–Altman agreement plot with proportional‐bias trend for intraoperatively treated versus TVS‐significant fibroid counts. Bland–Altman plot showing agreement between the preoperative TVS‐significant fibroid count and the intraoperatively treated fibroid count (N = 67). The y‐axis depicts the paired difference (intraoperative − TVS‐significant), and the *x*‐axis depicts the mean of the paired counts. The solid horizontal line indicates the median paired difference (median bias), and dashed lines indicate the 2.5th and 97.5th percentiles of the paired differences. The solid sloped line represents a linear trend fitted to the paired differences versus the paired mean to visualize proportional bias.

Bland–Altman analysis demonstrated a median bias of 0 fibroids (intraoperative − TVS‐significant), with 2.5th–97.5th percentile limits ranging from −1.00 to +2.00 fibroids, indicating substantial individual‐level variability despite frequent ties (Figure 2). Proportional bias was evident, with larger positive paired differences at higher average fibroid counts (Spearman *ρ* = 0.40, *P* = .00079; Kendall *τ*‐*b* = 0.36, *P* = .00078). Moreover, the magnitude of disagreement increased with fibroid burden (|difference| versus paired mean: Spearman *ρ* = 0.61, *P* < 1 × 10^−7^; Kendall *τ*‐*b* = 0.55, *P* < 1 × 10^−6^), consistent with increasing variability at higher counts.

### Subgroup Analysis: Multifibroid Disease

In the multifibroid subgroup (≥2 clinically significant fibroids on TVS; n = 23), TVS identified a mean of 2.74 ± 0.75 fibroids compared with 3.00 ± 1.38 treated intraoperatively. The mean paired difference was +0.26 (95% CI −0.17 to 0.69) and was not statistically significant (Wilcoxon *P* = .266). In this subgroup, intraoperative counts exceeded TVS counts in 8 patients (34.8%), were identical in 11 (47.8%), and were lower in 4 (17.4%).

### Correlative Analyses

The number of intraoperatively treated fibroids showed a strong monotonic association with the TVS‐significant fibroid count (Spearman *ρ* = 0.72, *P* < .001; n = 67). Categorized agreement analysis (1, 2, and ≥3 fibroids) demonstrated an exact agreement in 73.1% of cases. The linearly weighted Cohen's kappa was 0.61 (95% CI 0.42–0.76), indicating substantial agreement between preoperative TVS and intraoperative findings. Treated fibroid count showed a modest association with total ablation time (*ρ* = 0.26, *P* = .033; n = 66). No statistically significant associations were observed between treated fibroid count and changes in hemoglobin, leukocytes, or C‐reactive protein between postoperative day 1 and day 2 (Hb_Diff: *ρ* = 0.18, *P* = .17, n = 63; Leuko_Diff: *ρ* = −0.06, *P* = .66, n = 62; CRP_Diff: *ρ* = −0.10, *P* = .44, n = 61).

### Safety Outcomes

No intraoperative conversions, device malfunctions, or major complications were recorded. Two patients (3%) experienced transient postoperative fever that resolved with conservative management. One patient required readmission due to fibroid expulsion. Importantly, no increase in complication rate was observed with higher fibroid counts, including in the multifibroid subgroup.

## Discussion

In this two‐center cohort, preoperative transvaginal sonography (TVS) yielded numerically lower counts of clinically significant fibroids compared to the number ultimately selected for treatment intraoperatively by transcervical RFA. The mean paired bias was +0.30 fibroids (intraoperative minus TVS). However, the effect was clearly subset‐driven: intraoperative counts exceeded TVS‐significant counts in 28% of patients, were identical in 66%, and were lower in only 6%. This distribution explains why the median paired difference is 0 while a statistically significant directional bias remains.

Furthermore, when evaluating patients with multifibroid disease—strictly defined a priori based on preoperative TVS to ensure an unbiased comparison—the mean paired difference (+0.26 fibroids) was not statistically significant. This indicates that the numerical underrepresentation observed in the overall cohort is not purely a systematic limitation in patients preoperatively identified with multiple lesions but rather reflects individual intraoperative expansions of the treatment strategy.

Our findings align with the broader imaging‐mapping literature showing that TVS performs well as a first‐line tool for confirming fibroid presence. Intraoperative ultrasound studies in minimally invasive fibroid surgery report that real‐time procedural mapping can reveal additional intramural or smaller lesions not appreciated preoperatively. The present data refine this for transcervical RFA workflows: the dominant signal is not pervasive diagnostic failure, but substantial agreement coupled with a clinically relevant minority in whom intraoperative mapping identifies additional actionable lesions. This distinction matters for counseling and planning—TVS remains highly informative for most patients, while a subset may experience an expanded treatable lesion set at the time of the procedure.^10^, ^11^

Intraoperative treated fibroid count correlated strongly with TVS‐significant count (Spearman *ρ* = 0.72, *P* < .001), indicating preserved rank‐order agreement between modalities. In contrast, the relationship between fibroid count and procedural time was modest: ablation time correlated weakly with treated fibroid number (Spearman *ρ* = 0.26, *P* = .033). These findings confirm that procedural duration is influenced by factors beyond fibroid count alone (eg, size, location, access, and strategy).

Laboratory markers did not support a clinically meaningful increase in inflammatory response or blood‐loss surrogates with multifibroid treatment. Overall, these data do not indicate a systematic physiologic penalty—within the limits of available laboratory completeness—associated with treating multiple fibroids. However, these laboratory comparisons are strictly exploratory and limited by the sample size, precluding definitive conclusions regarding perioperative morbidity.

Strengths of this study include the two‐center cohort, paired within‐patient comparison, and analytic choices that are appropriate for discrete, non‐normally distributed counts. Several limitations exist. First, the intraoperative comparator reflects therapeutically selected fibroids rather than a pathological ground truth; therefore, the study quantifies the discrepancy in actionable burden rather than the absolute diagnostic sensitivity of TVS. Second, the classification of “clinically significant” on TVS reflects real‐world clinical judgment by attending sonographers without a blinded central imaging review. Third, the retrospective nature limits standardized lesion‐level documentation (eg, precise volume or exact topographical mapping), which would enable mechanistic inferences about which lesion phenotypes are most frequently reclassified intraoperatively. Finally, this study does not assess long‐term symptom resolution, reintervention rates, or clinical failure. Consequently, inferences regarding the direct impact of preoperative numerical discrepancies on long‐term clinical outcomes cannot be drawn.

## Conclusion

Preoperative TVS demonstrates substantial agreement with the intraoperatively treated fibroid count during transcervical RFA. While a systematic underrepresentation was not confirmed in the preoperatively defined multifibroid subgroup, clinicians should anticipate a potential intraoperative expansion of the treatable lesion burden in approximately one‐quarter of cases. TVS remains a highly reliable first‐line modality for patient selection and baseline characterization, but procedural planning should incorporate the flexibility to treat additional lesions identified during real‐time intrauterine mapping.

## Supporting information


**Supplemental Figure 1.** Distribution of paired differences (intraoperative − TVS‐significant fibroid count). Bar plot of the discrete paired differences between intraoperatively treated fibroid count and TVS‐significant fibroid count in the combined cohort (N = 67), highlighting the frequency of ties (difference = 0) and the subset of patients with positive differences.

## Data Availability

The data that support the findings of this study are available from the corresponding author upon reasonable request.
